# Recently formed Antarctic lakes host less diverse benthic bacterial and diatom communities than their older counterparts

**DOI:** 10.1093/femsec/fiad087

**Published:** 2023-07-29

**Authors:** Jan Kollár, Kateřina Kopalová, Jan Kavan, Kristýna Vrbická, Daniel Nývlt, Linda Nedbalová, Marek Stibal, Tyler J Kohler

**Affiliations:** Faculty of Science, Department of Ecology, Charles University, Viničná 7, Prague 2, CZ-12844, Czech Republic; Faculty of Science, Department of Ecology, Charles University, Viničná 7, Prague 2, CZ-12844, Czech Republic; Polar-Geo-Lab, Faculty of Science, Department of Geography, Masaryk University, Kotlářská 2, Brno, CZ-61137, Czech Republic; Alfred Jahn Cold Regions Research Centre, University of Wroclaw, pl. Uniwersytecki 1, Wroclaw 50-137, Poland; Faculty of Science, Department of Ecology, Charles University, Viničná 7, Prague 2, CZ-12844, Czech Republic; Polar-Geo-Lab, Faculty of Science, Department of Geography, Masaryk University, Kotlářská 2, Brno, CZ-61137, Czech Republic; Faculty of Science, Department of Ecology, Charles University, Viničná 7, Prague 2, CZ-12844, Czech Republic; Faculty of Science, Department of Ecology, Charles University, Viničná 7, Prague 2, CZ-12844, Czech Republic; Faculty of Science, Department of Ecology, Charles University, Viničná 7, Prague 2, CZ-12844, Czech Republic

**Keywords:** 16S rDNA, climate change, cryosphere, cyanobacteria, diatom, glacier

## Abstract

Glacier recession is creating new water bodies in proglacial forelands worldwide, including Antarctica. Yet, it is unknown how microbial communities of recently formed “young” waterbodies (originating decades to a few centuries ago) compare with established “old” counterparts (millennia ago). Here, we compared benthic microbial communities of different lake types on James Ross Island, Antarctic Peninsula, using 16S rDNA metabarcoding and light microscopy to explore bacterial and diatom communities, respectively. We found that the older lakes host significantly more diverse bacterial and diatom communities compared to the young ones. To identify potential mechanisms for these differences, linear models and dbRDA analyses suggested combinations of water temperature, pH, and conductivity to be the most important factors for diversity and community structuring, while differences in geomorphological and hydrological stability, though more difficult to quantify, are likely also influential. These results, along with an indicator species analysis, suggest that physical and chemical constraints associated with individual lakes histories are likely more influential to the assembly of the benthic microbial communities than lake age alone. Collectively, these results improve our understanding of microbial community drivers in Antarctic freshwaters, and help predict how the microbial landscape may shift with future habitat creation within a changing environment.

## Teaser

While glacier recession creates new lakes in Antarctica, they host considerably less diverse bacterial and diatom communities than their older counterparts, but primarily not due to lake age alone.

## Introduction

The Antarctic Peninsula is one of the most rapidly changing regions on Earth (Vaughan et al. 2003, Turner et al. 2021, Flexas et al. 2022). Here, the physical feedbacks of climate warming have been well-documented over the last decades, especially in terms of atmospheric conditions, marine ecosystems, and glacier mass balance (Siegert et al. 2019, Engel et al. 2022). Yet, we are only beginning to understand how this shift may influence the biology of the Antarctic Peninsula’s diverse freshwater ecosystems. Freshwater habitats are abundant in the < 1% of Antarctica, which is deglaciated, and consists of streams, seepages, ponds, and lakes. Each of these habitat types represents an important hotspot of productivity and biodiversity in an otherwise inhospitable landscape (e.g. Kopalová et al. 2013, 2019), forming the basis of local limno-terrestrial foodwebs and performing biogeochemical cycling relevant at the landscape scale (Ellis-Evans 1996). However, to detect ecological responses of freshwater communities to ongoing climate change, it is first necessary to understand the factors responsible for their structure, which can then be used to make informed predictions about their subsequent trajectories.

Among other things, lakes are widely recognized as valuable models for the study of diversity and evolution (Laybourn-Parry and Pearce 2007, Vincent and Laybourn-Parry 2008), archives of past climate conditions (Sterken et al. 2012, Píšková et al. 2019, Čejka et al. 2020), and sentinels of current and future change (Adrian et al. 2009, Williamson et al. 2009). In Antarctica, it is expected that ponds and lakes will increase in their water temperature, with more ice-free days over the austral summer in the future (Quayle et al. 2002). These changes, often coupled with greater nutrient supply from enhanced hydrological connectivity with the surrounding watershed, will likely also lead to community shifts, including an increase in primary producer biomass (Quayle et al. 2002). On top of these changes, the creation of new habitats, including lakes and ponds, is anticipated with glacier retreat (Lee et al. 2017), as has already been observed in Antarctica (Kiernan and McConnell 2002, da Rosa et al. 2022) and Earth’s alpine regions (Carrivick and Tweed 2013). Yet, it is not clear how these “young” water bodies will compare with older ones in terms of their capacity to harbor local bacterial and microeukaryotic communities. Elsewhere, glacial and proglacial lakes exhibit predictable ontogenies (Sommaruga 2015), with the result that recently formed lakes can harbor different communities than older ones (Peter et al. 2018). Collectively, given the creation of new habitats and anticipated shifts in the existing ones, it is necessary to investigate the environmental drivers of community structure to understand how future conditions will influence Antarctic freshwaters.

Microbial communities dominate Antarctic freshwater habitats, often forming conspicuous, brightly colored “mats” covering the surface of lake sediments (Vincent et al. 1993). As a result of this microbial dominance, as well as the technological constraints of the last century, most biological work in Antarctic freshwaters has utilized microscopy to characterize communities. As a consequence, studies have also been biased toward organisms that are distinguishable under the microscope, such as the cyanobacteria, microalgae, protists, and meiofauna. Yet, nonphotosynthetic bacteria, which probably encapsulate the bulk of the diversity (both taxonomic and functional), have been underexplored (Franzmann 1996, Laybourn-Parry and Pearce 2016). To date, there are relatively few investigations on the controls of bacterial community structure in Antarctic lentic habitats (e.g. Archer et al. 2015, Zhang et al. 2015, Jackson et al. 2021), especially alongside more traditionally studied groups such as diatoms. As a result, we are still only beginning to understand the diversity of Antarctic freshwater microorganisms, let alone how these communities will shift in the future.

Here, we investigate the diverse ponds and lakes of Ulu Peninsula, on James Ross Island (JRI), north-eastern Antarctic Peninsula, to learn more about the drivers of benthic microbial community structure in these relatively pristine, but highly endangered, ecosystems. Specifically, we asked: (1) are there differences in the diversity and taxonomic composition of benthic microbial communities inhabiting lakes of different ages (originating decades versus millennia ago), and (2) what are the most important environmental drivers structuring these communities? To answer these questions, we characterized bacterial communities through 16S rDNA and diatom communities through light microscopy, the latter of these potentially serving as a proxy providing basal insight into diversity patterns among microeukaryotes. We hypothesized that older lakes should have greater diversity and distinct communities from younger lakes, primarily due to long-term successional patterns and associated physico-chemical conditions related to lake ontogeny (e.g. warmer temperatures, organic carbon accumulation, geomorphological stability, and so on).

## Materials and methods

### Site description

**Figure 1.**
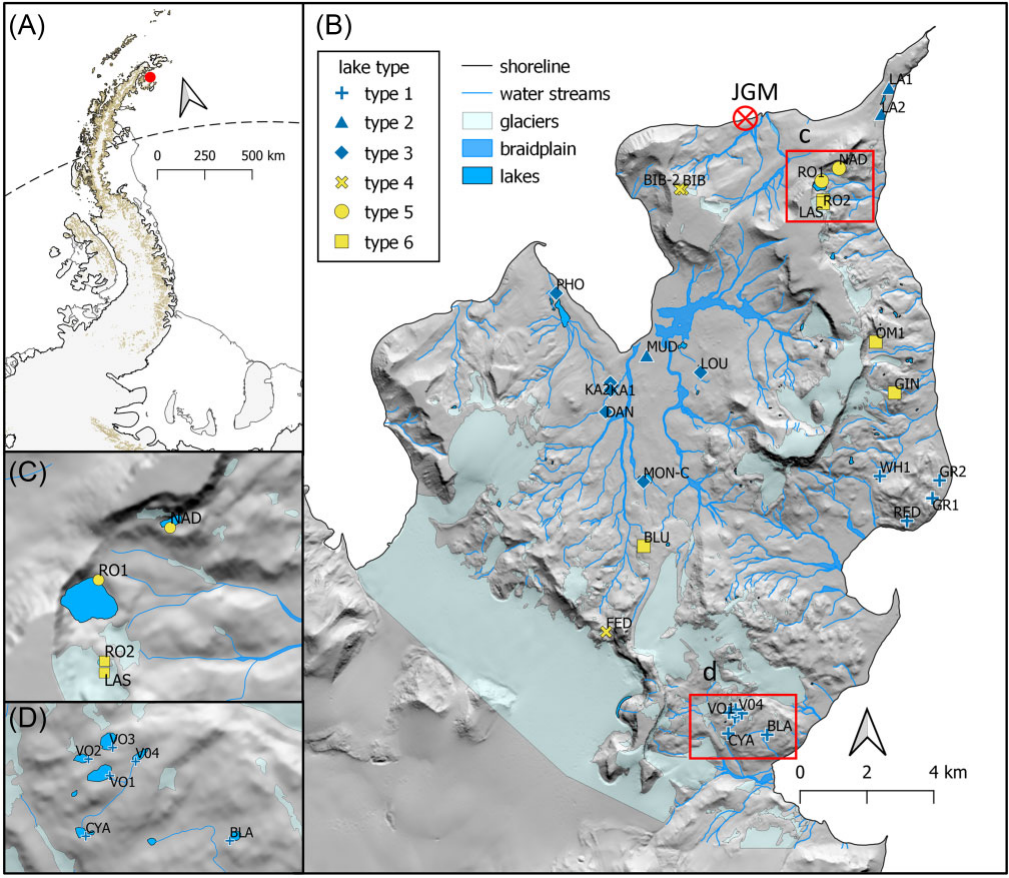
A detail of Ulu Peninsula, JRI, with its position within the Antarctic Peninsula **(A)**, as well as the position of the 29 sampled lakes, indicated (B)–(D). “JGM” in panel **(B)** indicates the position of the Czech Antarctic Johann Gregor Mendel Station. Lake age is designated by color, with “old” lakes in blue and “young” lakes in yellow, and lake type (1–6, from Nedbalová et al. 2013) is indicated by symbol shape.

JRI is located close to the northeastern extremity of the Antarctic Peninsula (Fig. 1A). It is nearly 2600 km^2^ in area and rises to a maximum elevation of 1630 m a.s.l. (at the Mount Haddington volcano). Summers are short (December–February) and the annual mean temperature at the Johann Gregor Mendel Czech Antarctic Station (situated near sea level, 63°48′02″S, 57°52′57″W; Fig. 1B) was −7°C between 2013 and 2016 (Ambrožová et al. 2019). Although deglaciation began 12.9 ka ago (Nývlt et al. 2014), more than 80% of the island is still covered by glaciers (Davies et al. 2013). The majority of the deglaciated area lies in the northern part of the island, on the Ulu Peninsula.

The lower-levelled coastal regions of the Ulu Peninsula were already deglaciated at the beginning of the Holocene (Nývlt et al. 2014), and currently, the deglaciated area occupies ca 250 km^2^ of the Ulu Peninsula (Jennings et al. 2021). The Ulu Peninsula itself is underlain by permafrost, with an active layer thickness around 60 cm at the peak of the austral summer (Kaplan Pastíriková et al. 2023). The geology of the Ulu Peninsula is characterized mainly by Cretaceous back-arc basin sedimentary rocks, mostly subglacial and partly effusive Neogene to Quaternary volcanic rocks, and less commonly intercalated or overlying sedimentary rocks (Mlčoch et al. 2020). The majority of precipitation falls as snow, and its distribution is strongly influenced by prevailing south to southwestern winds (Ambrožová et al. 2020, Kavan et al. 2020). Terrestrial vegetation cover is sparse, and dominated by lichens, mosses, cyanobacteria, and microalgae (Barták et al. 2015).

From a limnological perspective, the Ulu Peninsula hosts a diverse ensemble of lakes that were previously divided into six types based on their origin, bedrock geology, geomorphology, hydrological stability, and physical and chemical characteristics (Nedbalová et al. 2013). Briefly, these include (1) stable shallow lakes of higher-lying levelled surfaces, (2) shallow coastal lakes, (3) stable lakes in old moraines, (4) small unstable lakes in young moraines, (5) deep cirque lakes, and (6) kettle lakes (Nedbalová et al. 2013). Based on the deglaciation history briefly outlined above [see Note S1 (Supporting Information) for more details and references], the lakes which are located outside of the Neoglacial moraines (i.e. lake types 1–3, our “old” lakes) must be older than 2000 years. On the contrary, lakes associated with the deglaciation after the Neoglacial culmination (i.e. lake types 4–6, our “young” lakes) must be younger than ca 300 years, some of them originating during the last decades only.

In general, the young lakes tend to be deeper and somewhat shaded by the surrounding topography, leading to lower and more stable temperatures and longer durations with ice cover. Meanwhile, the old lakes tended to be shallower, are usually ice-free over summer, and have water temperatures more prone to fluctuation (up to nearly 20°C over a daily cycle; Nedbalová et al. 2013). In addition, young lakes commonly have more unstable shorelines, which along with longer durations of ice cover, can disturb the littoral habitat. As a result, they often differ from the old lakes by hosting less visually prominent cyanobacterial mats. Furthermore, while old lakes have more permanence resulting from enhanced geomorphological stability, they also tend to be shallower, and therefore, prone to periodic desiccation. A detailed description of these lakes, as well as greater discussion of the different lake types, can be found in Nedbalová et al. (2013).

### Field sampling

Microbial samples were collected from 29 lakes on the Ulu Peninsula (Fig. 1B–D; Table 1) between 8 and 17 February 2017. From each lake, the dominant substrate (surface sediment or stones) was sampled from the littoral zone within arm’s reach and at comparable depths to ensure comparability between lake types. Samples were taken with a steel scalpel or spoon (cleaned with ethanol between samples), transferred to a sterile centrifuge tube with LifeGuard (Qiagen, DE) conservation solution, and frozen as soon as possible (−20°C). In addition, physico-chemical parameters (water temperature, specific conductivity, and pH) were measured *in situ* using a Hanna Combo handheld device (Hanna Instruments, USA). Bulk water samples were taken via grab samples in clean plastic bottles, frozen, and sent to the T. G. Masaryk Water Research Institute (p.r.i., CZ) for analysis of their chemical properties, including concentrations of total phosphorus (P_tot_), dissolved organic carbon (DOC), total nitrogen (N_tot_), nitrate (NO_3_^−^), ammonium (NH_4_^+^), sulfate (SO_4_^2−^), fluoride (F^−^), chloride (Cl^−^), sodium (Na^+^), potassium (K^+^), calcium (Ca^+^), and magnesium (Mg^+^) ions (see analytical details in Roman et al. 2019).

**Table 1.** Location, sampling time (all in 2017), and the most relevant characteristics of the JRI lakes. The information on lake type, bedrock, age, and lake area were extracted from Nedbalová et al. (2013). Samples analyzed for diatoms are indicated by an asterisk (n.a. = not available).

		Latitude	Longitude	Altitude	Sampling time				Lake area	Temperature		Conductivity
Lake	Code	(DD)	(DD)	(m a.s.l.)	(HH:MM, DD.MM.)	Type	Bedrock	Age	(m^2^)	(°C)	pH	($\mu$S cm^−1^)
Vondra 1	VO1*	−63.9613	−57.9028	229	14:20, 09.02.	1	V	Old	18 050	13.0	9.6	56
Vondra 4	VO4*	−63.9603	−57.8986	221	16:10, 09.02.	1	V	Old	6077	2.0	9.1	45
White	WH1*	−63.8971	−57.8124	65	12:15, 13.02.	1	V	Old	6662	8.2	7.7	128
Green 1	GR1*	−63.9033	−57.7805	65	14:00, 13.02.	1	V	Old	4220	6.8	8.8	97
Green 2	GR2*	−63.8985	−57.7761	40	14:40, 13.02.	1	V	Old	2970	7.9	9.2	176
Red	RED	−63.9093	−57.7962	65	13:00, 13.02.	1	V	Old	4324	7.4	8.9	265
Cyanobacterial	CYA*	−63.9657	−57.9069	185	13:45, 09.02.	1	V	Old	6944	15.7	9.2	84
Black	BLA	−63.9661	−57.8833	166	16:40, 09.02.	1	V	Old	5363	12.4	9.5	121
Vondra 3	VO3*	−63.9593	−57.9023	235	15:45, 09.02.	1	V	Old	13 300	10.9	9.0	55
Vondra 2	VO2*	−63.9601	−57.9063	228	15:00, 09.02.	1	V	Old	5977	11.1	9.0	49
Lachman 2	LA2*	−63.8000	−57.8086	13	19:15, 15.02.	2	G	Old	14 670	3.7	9.3	948
Lachman 1	LA1*	−63.7930	−57.8038	9	18:45, 15.02.	2	G	Old	29 670	5.2	7.2	411
Muddy	MUD	−63.8639	−57.9534	4	17:30, 12.02.	2	C	Old	10 940	7.9	7.9	376
Phormidium	PHO*	−63.8468	−58.0078	10	16:30, 12.02.	3	C+G	Old	158 100	7.1	8.2	268
Monolith C	MON-C*	−63.8978	−57.9565	67	09:40, 13.02.	3	C+G	Old	93 050	4.3	7.6	83
Katia 2	KA2*	−63.8711	−57.9757	38	18:30, 12.02.	3	C	Old	1518	6.8	7.8	198
Dan	DAN	−63.8789	−57.9788	41	19:15, 12.02.	3	C	Old	13 280	6.1	7.9	142
Louže	LOU*	−63.8687	−57.9207	40	12:20, 17.02.	3	n.a.	Old	12 150	4.9	8.0	552
Katia 1	KA1	−63.8731	−57.9758	36	18:55, 12.02.	3	C	Old	3289	5.3	7.8	149
Bibby	BIB*	−63.8195	−57.9300	250	16:00, 12.02.	4	M	Young	7439	2.1	9.2	29
Federico	FED	−63.9379	−57.9806	387	19:30, 08.02.	4	M	Young	8269	1.4	10.4	59
Bibby 2	BIB-2	−63.8195	−57.9300	250	16:00, 12.02.	4	M	Young	7439	2.1	9.2	29
Rožmberk	RO1*	−63.8177	−57.8455	201	16:00, 15.02.	5	M	Young	88 390	0.9	9.6	75
Naděje	NAD*	−63.8144	−57.8349	236	18:00, 15.02.	5	M	Young	8147	0.6	8.1	56
Blue–green	BLU*	−63.9151	−57.9568	184	n.a., 08.02.	6	M	Young	3806	5.5	7.8	91
Rozkoš	RO2*	−63.8230	−57.8448	260	17:00, 15.02.	6	M	Young	1944	0.9	8.8	77
Omega 1	OM1*	−62.8611	−57.8139	257	17:30, 13.02.	6	M	Young	24 320	0.6	9.2	54
Ginger	GIN*	−63.8748	−57.8028	197	16:10, 13.02.	6	M	Young	12 170	1.4	8.7	31
Láska	LAS*	−63.8237	−57.8448	269	16:30, 15.02.	6	M	Young	1455	1.1	9.3	63

Bedrock explanation: C = Late Cretaceous marine sediments, G = Miocene–Holocene glacial sediments composed mostly of local volcanic rocks and marine Cretaceous sediments, with minor admixture of Antarctic Peninsula derived igneous and metamorphic rocks, M = recent glaciogenic (morainic) sediments with present glacier ice composed mostly of local volcanic rocks, with some admixture of marine Cretaceous sediments, V = Neogene volcanic rocks consisting mostly of hyaloclastite breccias, tuffs, and subaerial basalts.

### DNA extraction, amplification, and sequencing

In the laboratory, ca 0.5 g of the sampled material was removed from tubes with flame-sterilized forceps or spatulae and DNA was extracted using a DNeasy PowerLyzer Powersoil Kit (Qiagen) following the manufacturer’s instructions. The resulting extract was quantified using a Qubit™ 4 Fluorometer with Qubit™ 1× dsDNA HS Assay Kit (Invitrogen™, USA; DNA concentration range = 4–27.2, median = 21.6 ng $\mu$l^−1^), and subsequently processed as in Kohler et al. (2020) and Vinšová et al. (2022), using samples of known mine bacterial community as a positive control. Briefly, 253 bp fragments of V4 16S rDNA region were PCR-amplified using the modified primers originally designed by Caporaso et al. (2011), 515F (GTGYCAGCMGCCGCGGTAA; Parada et al. 2016) and 806R (GGACTACNVGGGTWTCTAAT; Apprill et al. 2015). Sample-specific barcode tags were attached to the forward primer and 28-cycle PCR was performed using the HotStarTaq Plus Master Mix Kit (Qiagen). PCR was set as follows: initial melting step at 94°C for 3 min, followed by 28 cycles of 94°C for 30 s, 53°C for 40 s, and completed by 72°C for 1 min. After amplification, PCR products were checked in 2% (w/v) agarose gel. Samples were pooled together in equal proportions based on their molecular weight and DNA concentrations. Pooled samples were purified using Agencourt AMPure XP paramagnetic beads (Beckman Coulter, USA). The pooled and purified PCR products were used to prepare Illumina DNA library and sequenced at MR DNA (www.mrdnalab.com, Shallowater, TX, USA) on the Illumina MiSeq platform in a 2 × 250 bp setting.

### Bioinformatic analyses

Sequence data were processed in QIIME2 v. 2022.2.0 (Bolyen et al. 2019). The paired-end reads were demultiplexed and primers removed using the QIIME2 plugin *cutadapt* v. 2021.11 (Martin 2011). Denoising into amplicon sequence variants (ASVs) was performed using DADA2 (Callahan et al. 2016) as implemented in QIIME2, truncating and trimming the sequences based on the 25th percentile quality score (threshold = 30). The DADA2 pipeline incorporates basic quality-score-based filtering, correction of errors in marginal sequences, removal of chimeric sequences, removal of singletons, joining the denoised paired-end reads, and dereplication of the sequences.

Taxonomy was assigned through the machine learning-based Naïve Bayes classifier (Bokulich et al. 2018, Robeson et al. 2021) trained on the V4 16S rDNA sequences (i.e. 253 bp trimmed according to the 515F/806R primer pair) from the SILVA rRNA database v. 13.8 (Quast et al. 2013). Based on the assigned taxonomy, chloroplasts, mitochondria, Archaea (since they were of negligible diversity and abundance), and common laboratory contaminants (i.e. 12 ASVs with a total frequency of 12 800 sequences assigned to genera *Brevundimonas, Corynebacterium, Escherichia, Haemophilus*, and *Staphylococcus* with confidence > 95%) were removed from the dataset, the latter determined using published lists of known contaminants (Salter et al. 2014, Glassing et al. 2016) and our own internal database.

Following Peter et al. (2018), chloroplast sequences separated above were taxonomically reassigned with the Naïve Bayes classifier trained on the V4 16S rDNA sequences from the devoted PhytoREF database (Decelle et al. 2015) to increase taxonomic resolution. The PhytoREF is a curated database of 6490 16S rDNA plastidial sequences covering all known major photosynthetic lineages of the eukaryotic tree of life. However, when interpreting the results based on plastidial 16S rDNA, we must be aware that (1) the majority of sequences in the database are from marine microalgae, (2) the plastidial 16S rDNA is more variable compared to the bacterial one and 515F/806R thus covers only a fraction of the present eukaryotic diversity, (3) the 16S rDNA have lower taxonomic resolution than other plastidial genes, such as *rbc*L, and (4) there is considerable taxon-specific variability in a number of chloroplasts (and thus also plastidial DNA copies) per cell across the eukaryotic tree of life. Nevertheless, the gene may still prove useful in a cautious exploration of the eukaryotic primary producer communities at higher taxonomic levels.

### Morphological analysis of diatom communities

Based on the initial investigation of the plastidial 16S rDNA (see above), diatoms (Bacillariophyta) proved to be the most diverse, and generally most prevalent, eukaryotic primary producers in the lake sediments of the Ulu Peninsula (see the section “Results”). Because of this, the ability of diatoms to potentially serve as a proxy for other protist groups, and to link our research with past biomonitoring and paleoecological efforts in the region, we decided to further investigate the taxonomic composition of diatom communities under light microscope. To this end, we prepared the samples following Van der Werff (1955), cleaning them with 37% H_2_O_2_ and heating them at 80°C for an hour. Oxidation of organic matter was completed by the addition of KMnO_4_. The oxidized material was then centrifuged (10 min at 3700 *g*), diluted with distilled water, dried on microscope cover slips, and mounted in Naphrax® synthetic resin. For each sample, 400 diatom frustules in random transects were identified to the species level at 1000× magnification under oil immersion using an Olympus BX43 light microscope with Nomarski contrast and an Olympus DP27 camera. The taxonomic identification is based primarily on the Iconographia Diatomologica vol. 24 (Zidarova et al. 2016) and the references therein.

### Statistical analyses

The *qiime2R* (v. 0.99; Bisanz 2018) R package was used to transfer data from QIIME2 to R (v. 4.2.1; R Core Team 2022), and subsequent statistical analyses were performed primarily utilizing functions within the *vegan* (Oksanen et al. 2020) and *phyloseq* (McMurdie and Holmes 2013) packages. To explore environmental gradients among lakes and lake types, we used principal component analysis (PCA) to visualize differences among sites in terms of their environmental characteristics. We included lake area (taken from Nedbalová et al. 2013), altitude, water temperature, pH, specific conductivity, P_tot_, and DOC, as well as concentrations of NH_4_^+^ and SO_4_^2−^ ions because the former was the only N species over the detection limit for most lakes (Table S1, Supporting Information), and the latter may inform about the potential for certain microbial metabolisms (e.g. sulfate reduction). The plot was generated using the autoplot() command in the *ggfortify* R package (Tang et al. 2016), with all variables mean-centered and standardized. Here and below, variable distributions were first assessed through histograms and log- or log_10_-transformed if necessary to create approximately normal distributions. Lastly, to prevent the removal of the BLU sample due to the missing values for water temperature, conductivity, and pH, these values were extracted from Nedbalová et al. (2013).

To investigate differences in and drivers of lake bacterial and diatom diversity, samples were rarified to a sampling depth of 52 626 sequences (based on a review of the feature table and plots of alpha diversity metrics against sampling depth; Figure S1, Supporting Information). Alpha diversity metrics were then computed (Observed number of bacterial ASVs/diatom morphospecies, Shannon Diversity Index, and Pielou’s Evenness Index; Shannon and Weaver 1949, Pielou 1966, DeSantis et al. 2006), and differences between lake age groups were tested with the Kruskal–Wallis test. In order to identify specific environmental variables explaining observed richness (i.e. the number of bacterial ASVs or diatom morphospecies), we first selected explanatory variables hypothesized to be important for community structure (nutrients were omitted due to being generally below detection limits), which included lake area, water temperature, conductivity, and pH. Collinearity among the selected variables was assessed with variance inflation factors (all <1.3). Linear mixed effects (lmer) models were created with the *lme4* R package (Bates et al. 2015) to enable the inclusion of lake age (young or old) as a random variable. Models were tested with and without lake age as a random variable, and the best of these models was chosen with compare_performance() command in the *performance* package (Lüdecke et al. 2021). Once the optimal random structure was identified (in the end, it was none), the best combination of fixed effects was isolated through backwards selection using the step() function. The model was then validated using the check_model() command, and results obtained using the summary() command.

In order to compare community structure among lakes, beta diversity metrics (Jaccard Distance, emphasizing presence/absence; Jaccard 1908; and Bray–Curtis Dissimilarity, emphasizing relative abundance; Sørensen 1948) were visualized through principal coordinates analysis (PCoA) ordinations. Differences in community structure between old and young lakes were statistically tested with a permutational multivariate analysis of variance (PERMANOVA; Anderson 2001) with 999 permutations using adonis2() in the *vegan* package. This was followed by a homogeneity of dispersion test (to investigate if old/young groupings have statistically dissimilar dispersions) conducted with the betadisper() function in *vegan*. To investigate individual taxa contributing to the differences between old and young lake communities, a multilevel pattern analysis was performed to identify the indicator taxa (Dufrêne and Legendre 1997, De Caceres and Legendre 2009) using the *indicspecies* package in R, as well as analysis of composition (ANCOM; Mandal et al. 2015) in QIIME2.

Lastly, to find the most parsimonious combination of environmental parameters explaining variability in bacterial and diatom communities across the Ulu Peninsula, we computed distance-based redundancy analysis (dbRDA) models similar to those implemented in Sakaeva et al. (2016) and Kohler et al. (2020). Briefly, dbRDA models were computed for both the bacterial and diatom datasets using Bray–Curtis distances (since Bray–Curtis explained the most variability in the PCoA analyses), and candidate models were constructed by including the same set of variables utilized in the linear models describing bacterial and diatom richness above (i.e. only environmental variables with variance inflation factor less than 1.3, including lake area, water temperature, pH, and conductivity). The best combination of variables for each dataset was then isolated through backward selection using the ordistep() function in *vegan*, and the significance of the full model, as well as individual terms, was assessed using the anova() function.

### Data and materials availability

Amplicon V4 16S rDNA data are available in the Sequence Read Archive (SRA; https://www.ncbi.nlm.nih.gov/sra) under the project identifier PRJNA975054. Key QIIME2 artifacts (including metadata tracking their provenance) are available in JK’s repository (https://www.researchgate.net/profile/Jan-Kollar-6). The diatom permanent slides are stored at the Department of Ecology, Charles University, Prague, Czech Republic.

## Results

### Environmental variation

**Figure 2.**
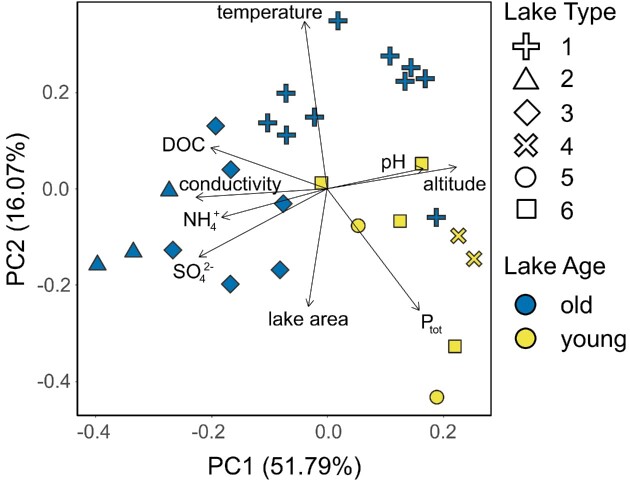
PCA comparing selected environmental variables of lakes. Lake age is designated by color, with “old” lakes in blue and “young” lakes in yellow, and lake type (1–6, from Nedbalová et al. 2013) is indicated by symbol shape.

For the 29 lakes sampled (Fig. 1B–D), a comprehensive set of environmental variables was assembled (Table 1; Table S1, Supporting Information) and analyzed through PCA (Fig. 2). Most observations for NO_3_^−^ and N_tot_ were below the detection limit, and thus omitted from further analyses. The same was true for many of the lakes in terms of NH_4_^+^ and P_tot_, and thus relationships with these variables should be interpreted with some caution. The PCA revealed that altitude and pH correlated positively, and conductivity negatively, with the first principal component suggesting that these variables explained most of the limnological variation in the area (51.8%). The second principal component correlated with water temperature and lake area, and explained 16.1% of the observed variation. Most of the old lakes were associated with lower altitudes, P_tot_, and pH values, while having greater conductivities, DOC, and temperatures compared with young lakes (Fig. 2; Table S2, Supporting Information).

### Taxonomic composition of the bacterial communities

The V4 16S rDNA amplicon sequencing resulted in a dataset of 3 418 992 sequences (ca between 60 000 and 190 000 per sample). Demultiplexing and DADA2 denoising resulted in 2 940 239 sequences (ca between 53 000 and 165 000 per sample) divided among 8063 ASVs. The sequences removed in the DADA2 pipeline included ca 64 000 sequences identified as chimeric. Taxonomy-informed filtering (i.e. removing chloroplasts, mitochondria, and Archaea) resulted in 2 804 367 sequences belonging to 7558 ASVs. The average number of sequences per sample equaled 94 294 ± 26 080. The total number of sequences per ASV ranged between 2 and 179 015 with a median of 26 and an interquartile range of 92 (i.e. 50% of all ASVs were observed with abundances between 9 and 101 sequences).

**Figure 3.**
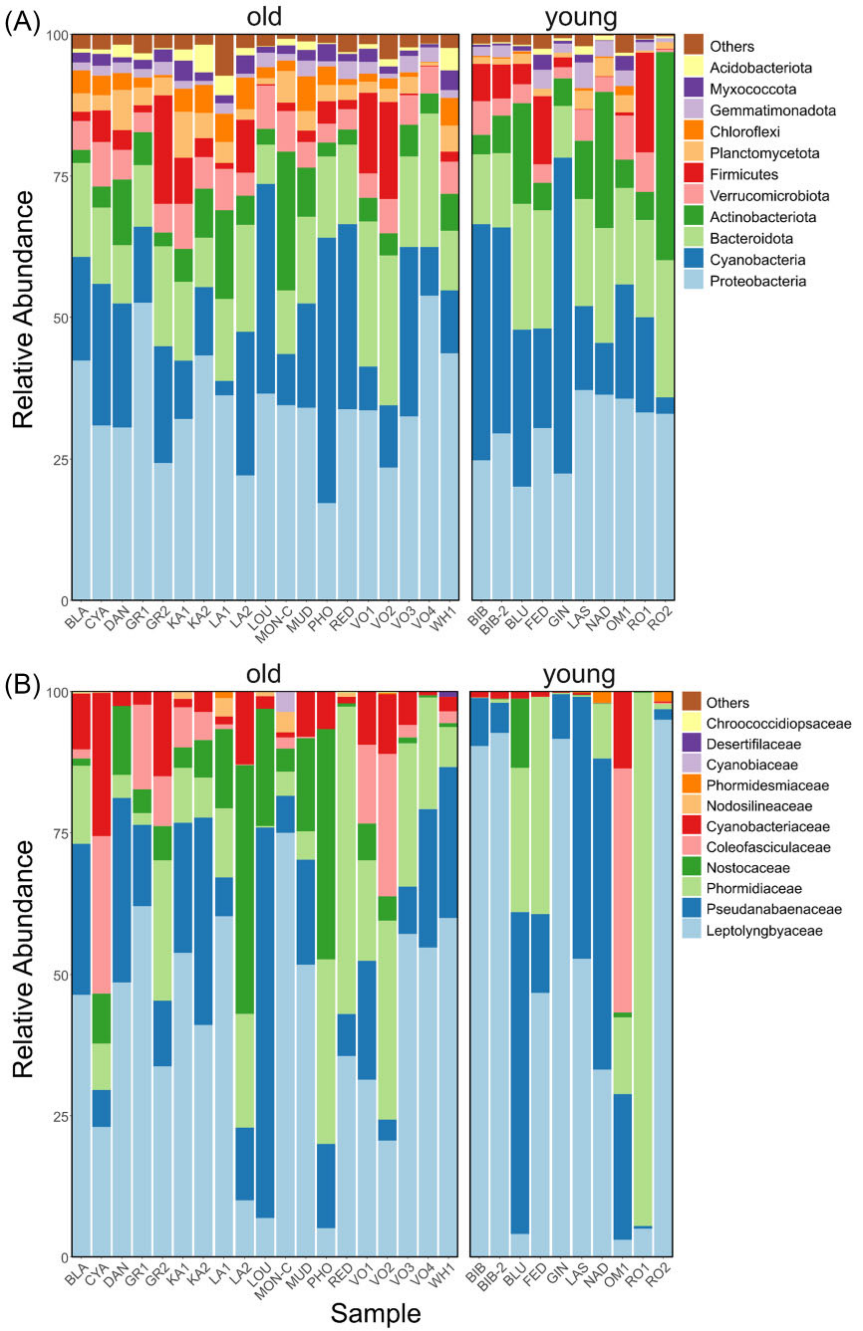
Sequence-based relative abundances of the most abundant bacterial phyla **(A)** and cyanobacterial families **(B)** in lake samples grouped by “old” (left) and “young” (right) ages.

A total of 7558 ASVs was assigned to 1317 genera. Of these, only 51 (i.e. 3.9%) were present in all 29 lakes including the cyanobacteria *Leptolyngbya, Pseudanabaena*, and *Tychonema*, the Gammaprotebacteria *Pseudomonas* and *Polaromonas*, and others such as *Ferruginibacter, Carnobacterium, Cryobacterium, Luteolibacter*, and *Gemmatimonas*. In 90% of the lakes, 103 bacterial genera were shared (7.8%) and 314 (23.8%) in 50% of the lakes. At the level of ASVs, 16 (0.2%) were shared across all of the lakes, 46 (0.6%) across 90% of the lakes, and 253 (3.3%) across 50% of the lakes. Cyanobacteria were among the most abundant bacterial groups with 615 885 sequences (i.e. 22.0% of the total) in 277 ASVs (i.e. 3.7%). Cyanobacterial families Leptolyngbyaceae, Phormidiaceae, and Pseudanabaenaceae were among the most abundant (in relative terms), along with Comamonadaceae and Pseudomonadaceae from the Gammaproteobacteria (Fig. 3). While the observed richness of cyanobacteria was between 10 and 69 ASVs per sample with an average of 33 ± 15 (median = 34, Q_1_ = 20, and Q_3_ = 42), for the bacterial community as a whole there were between 399 and 1911 ASVs per sample with an average of 870 ± 383 (median = 742, Q_1_ = 589, and Q_3_ = 1166). The noncyanobacterial portion of the bacterial community exhibited an average richness of 837 ± 371 ASVs per sample (median = 690, Q_1_ = 567, and Q_3_ = 1120).

### Diatoms and other microeukaryotic primary producers

To gain insight into the structure of the eukaryote photosynthetic community, an analysis of the plastidial V4 16S rDNA sequences was conducted. The results suggested that eukaryotic primary producers were represented mainly by Bacillariophyta (90 846 sequences in 51 ASVs), Eustigmatophyceae (6473 sequences in five ASVs), and Trebouxiophyceae (1270 sequences in 15 ASVs). All five ASVs of Eustigmatophyceae were identified as members of the genus *Nannochloropsis* with a confidence of 99% or higher (confidence values were computed by 100 times bootstrapping the 8-mers produced by machine learning Naïve Bayes classifier). Interestingly, in four lakes (FED, RO1, VO2, and MON) the Eustigmatophyceae reached >75% of the eukaryotic primary producers, surpassing even the usually dominant diatoms.

**Figure 4.**
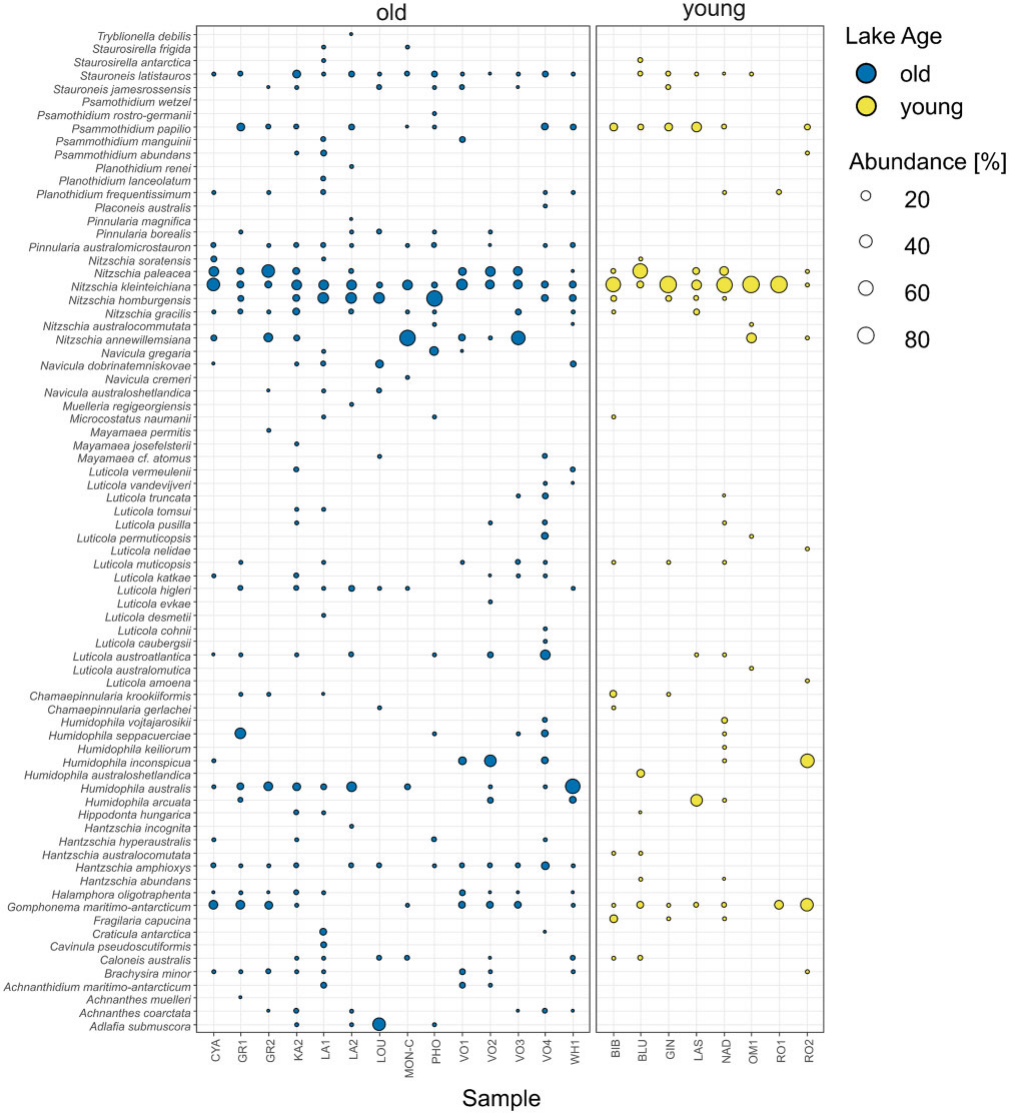
Relative abundance and distribution of diatom morphospecies (*y*-axis) in samples (*x*-axis) grouped according to lake age. “Old” lakes are colored in blue, and “young” lakes are colored yellow. Symbol size is proportionate to the relative abundance of a given taxon in the respective sample.

Based on light microscopy, a total of 76 diatom morphospecies was identified (Fig. 4) in the 22 analyzed samples (Table 1). Species richness ranged between 3 (lake “RO1”) and 29 (“LA1” and “KA2”) per sample with an average of 16 ± 7 species per sample. The most abundant species belonged to the genera *Nitzschia* (*N. kleinteichiana* found in 22 samples and representing 28.4% of all counted diatoms, *N. paleacea* found in 14 and representing 9.9%, *N. homburgensis* found in 12 and representing 8.7%, and *N. annewillemsiana* found in eight and representing 7.9%) followed by *Gomphonema* (*G. maritimo-antarcticum* found in 15 and representing 6.0%), and *Humidophila* (*H. australis* found in nine and representing 5.9%, and *H. inconspicua* found in six and representing 4.9%; Fig. 4).

### Drivers of microbial diversity

**Figure 5.**
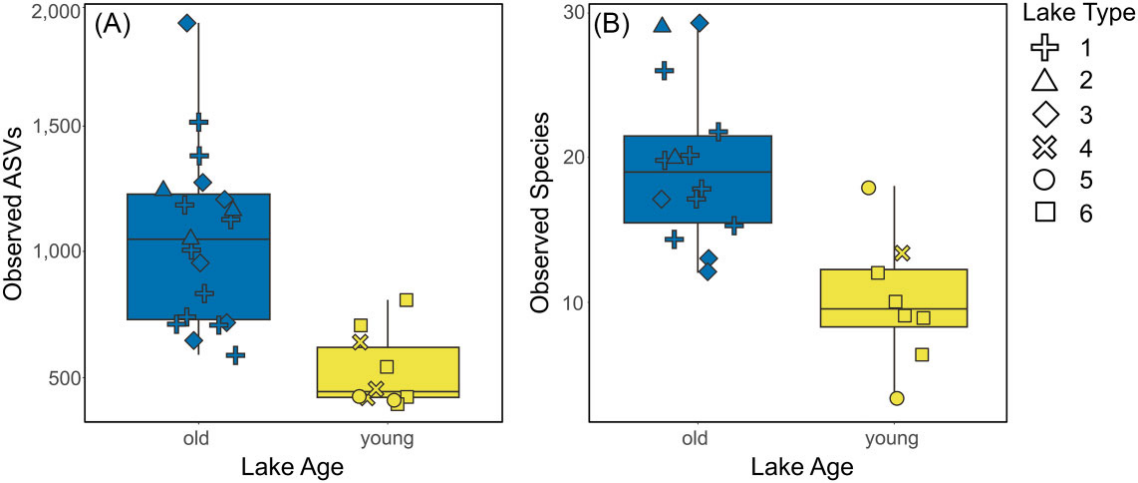
Differences in observed richness between old (blue) and young (yellow) lakes for bacteria (**A**; as number of ASVs) and diatoms (**B**; as number of morphospecies). Symbol shape indicates which lake type a sample originates from (1–6; Nedbalová et al. 2013). Old lakes have significantly greater richness values than young lakes for both bacteria and diatoms (Kruskal–Wallis test: *H* = 10.91 and 10.57, *P* ≈ .001 and .001, respectively).

Rarefaction of the samples to a sampling depth of 52 626 sequences per sample allowed us to retain all 29 samples for bacterial diversity analyses (Table 1) with both richness and evenness saturating at far lower sampling depths (Figure S1, Supporting Information). For both bacteria and diatoms, alpha diversity was significantly higher in old lakes compared to young both in terms of richness and evenness (Table S3, Supporting Information; Kruskal–Wallis: *H* between 6.14 and 15.57, all *P* < .02), and was approximately twice as high on average in the case of the former (Fig. 5). Bacteria had an average observed richness of 1053 ± 340 ASVs per sample in the old lakes while the young lakes hosted on average 524 ± 147 ASVs per sample. Cyanobacteria alone exhibited an average observed richness of 40.7 ± 12.4 ASVs per sample in the old lakes while the young lakes hosted on average 19.1 ± 6.2 ASVs per sample (*H* = 15.41, *P* < .001). Proportions of the cyanobacterial sequences per sample were not significantly different between young and old lakes (*H* = 0.61, *P* = .44). Concerning diatoms, an average of 19.4 ± 5.5 morphospecies per sample was observed in the old lakes while the young lakes hosted on average 10.0 ± 4.5 morphospecies.

To investigate mechanistic drivers of both bacterial and diatom diversity, we further created linear models to identify variables significantly associated with observed richness. For bacterial diversity, the most parsimonious model explained 54.93% (*R^2^* statistic) of the variation (*F* = 8.62, *P* < .001) and included pH (*P* = .002), water temperature (0.002), conductivity (0.051) and lake area (0.054). However, only water temperature and pH were statistically significant, and were correlated with changes in diversity (temperature positively, pH negatively). For diatom diversity, the best model included pH (*P* = .029), water temperature (0.047), lake area (0.101), and conductivity (0.138), and explained 34.41% (*F* = 3.76, *P* = .023). Again, only pH and water temperature were significantly associated with changes in diversity (temperature positively, pH negatively).

**Figure 6.**
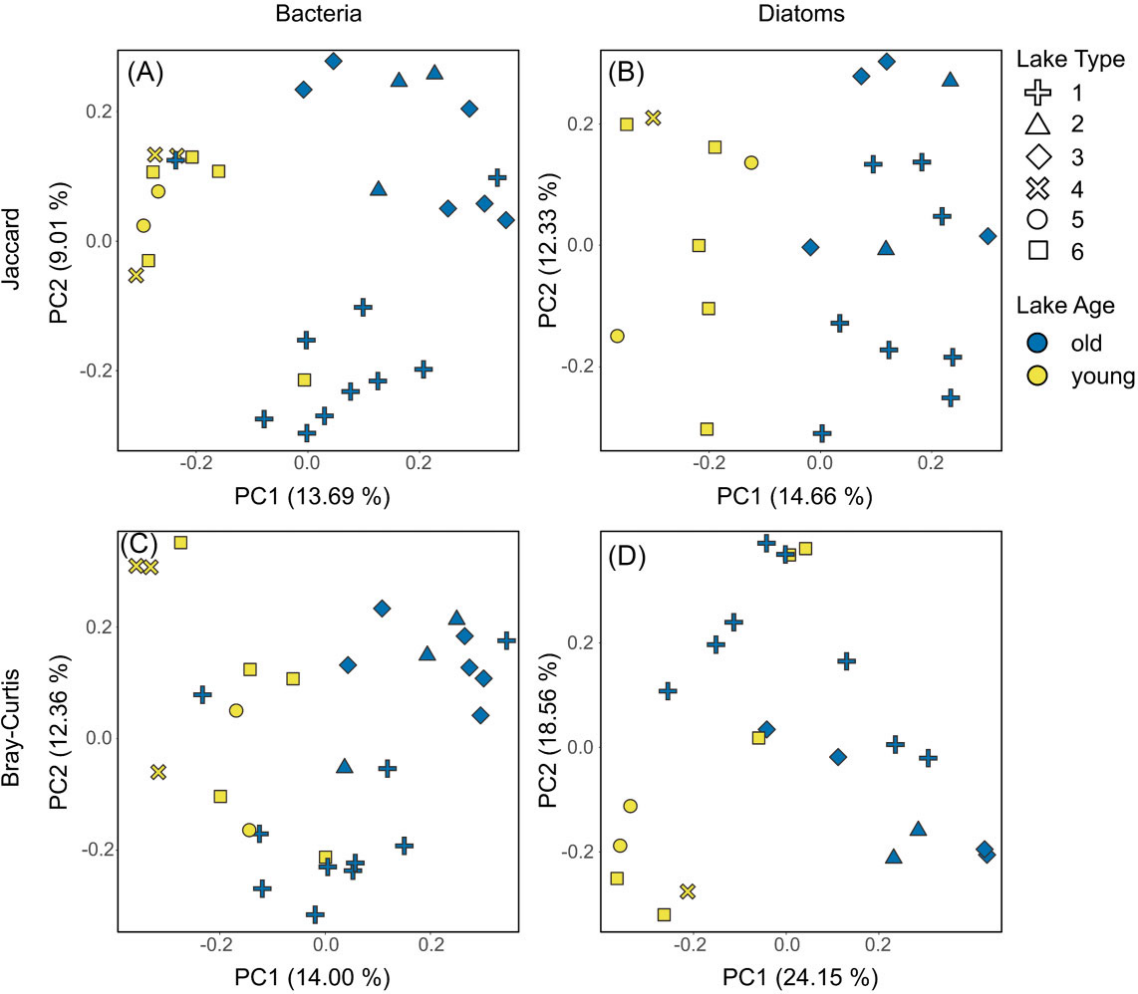
PCoA ordinations of the microbial communities based on Jaccard Distances (top, **A** and **B**) and Bray–Curtis Dissimilarities (bottom, **C** and **D**), with bacterial communities on left (**A** and **C**) and diatom communities on right (**B** and **D**). Colors correspond to lake age (with old lakes in blue and young lakes in yellow), and shapes correspond to the six lake types (*sensu* Nedbalová et al. 2013). Differences between old and young lakes were significant for all four analyses when tested with PERMANOVA.

To investigate patterns in beta diversity, bacterial and diatom communities were further plotted onto PCoA ordinations to visualize community differences between sites, using both Jaccard (emphasizing individual taxa) and Bray–Curtis (emphasizing relative abundances) distance matrices (Fig. 6). Consistent with the results for alpha diversity, we found a clear separation between the communities inhabiting old versus young lakes, which was statistically significant for all comparisons when tested with PERMANOVA (Fig. 6; *F* between 2.07 and 3.13, all *P* ≤ .002). In addition, the separation was not caused by differences in the dispersion of old versus young lake communities (*F* between 1.19 and 2.36, *P* between .115 and .291).

To investigate which taxa were primarily responsible for differences between lake age groups, we conducted multilevel pattern (i.e. indicator species) analyses to identify indicator taxa characteristic of young and old lake bacterial and diatom communities. For bacteria, the analysis identified 228 ASVs as significant (*P* ≤ .05) indicators of the old lakes, while 174 ASVs were indicative of young ones. On average, 15.4 ± 7.1% of reads found in the old lakes belong to the old lake indicators while in the young lakes, the proportion of the young lake indicator reads is 36.4 ± 17.4%. Among the indicators of the old lakes, 43 belonged to Alphaproteobacteria, 37 to Bacteroidota, 26 to Gammaproteobacteria (11 of these were Comamonadaceae), and 13 to Cyanobacteria. Overall, three cyanobacterial ASVs assigned to genera *Leptolyngbya* and *Pseudanabaena* showed the strongest association to the old lakes (*IndVal.g* statistic 0.961 and 0.914 for the former, and 0.933 for the latter). These were followed by one ASV of Alphaproteobacteria assigned to the genus *Polymorphobacter* (0.903) and one ASV of Myxococcota assigned to the genus *Aetherobacter* (0.896). Among the indicators of the young lakes, 39 were Bacteroidota, 29 Alphaproteobacteria, 24 Gammaproteobacteria with 11 Comamonadaceae, and six Cyanobacteria. The most represented genera were *Ferruginibacter* and *Fimbriiglobus* with five ASVs each, *Polaromonas, Sphingomonas*, and *Sediminibacterium* with three ASVs each, and *Pedobacter, Luteolibacter*, and *Rudanella* with two ASVs each. Among the ASVs exhibiting the strongest association to the young lakes were one cyanobacterial ASV identified as *Chamaesiphon* (*IndVal.g* = 0.946), one ASV of *Pedobacter* (0.944), one ASV of *Sphingomonas* (0.941), and one ASV of *Cryobacterium* (0.913). The parallel ANCOM analysis identified three ASVs (the same *Chamaesiphon, Pedobacter*, and *Sphingomonas* ASVs as above) significantly more abundant in the young lakes. There, these ASVs were observed in relative abundances reaching 2.8% (i.e. 2935 sequences, medians for the three ASVs are between 36 and 276 sequences per sample). In the old lakes, both *Pedobacter* and *Sphingomonas* indicator ASVs reached their maximal relative abundance in lake Vondra 4 (VO4; 0.6% and 0.2%, respectively). On average, 78.3 ± 5.2% of the old lake indicator ASVs represented “rare” taxa (arbitrarily defined as ASVs with < 0.1% of total community relative abundance; Lynch and Neufeld 2015). In the young lakes, the “rare” taxa represented 67.9 ± 9.1% of the young lake indicator ASVs.

For diatoms, the multilevel pattern analysis identified *Hantzschia amphioxys* (*IndVal.g* = 0.926), *Humidophila australis* (0.845), *Pinnularia australomicrostauron* (0.845), *Halamphora oligotraphenta* (0.802), and *Luticola higleri* (0.707) as indicator species of the old lakes while *N. kleinteichiana* (0.867) and *Fragilaria capucina* (0.612) were significantly associated with the young lakes. Finally, ANCOM identified one species of diatom that was differentially abundant between young and old lakes, *H. amphioxys*, which was observed in old lakes in relative abundances up to 11.8% (median 1.9%) per sample while entirely absent in young ones.

**Figure 7.**
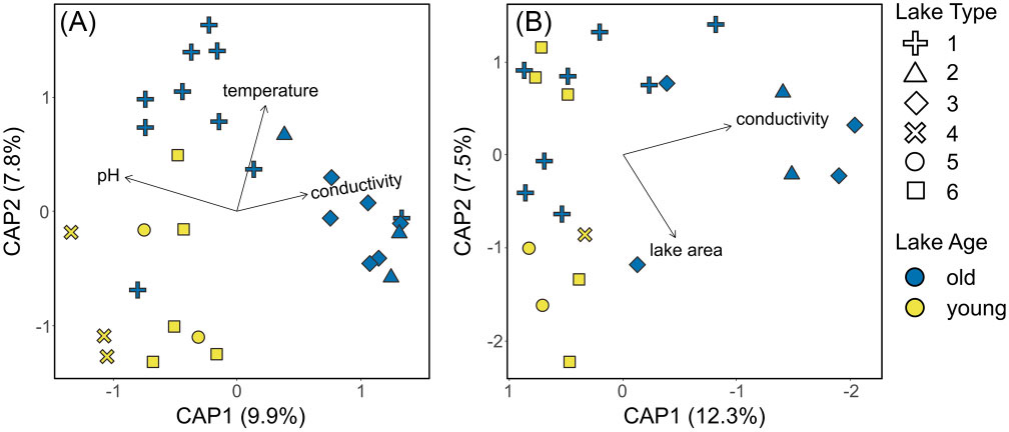
Distance-based redundancy analysis (dbRDA) based on Bray–Curtis distances computed for bacterial **(A)** and diatom **(B)** communities. Colors indicate lake age (young/old as yellow/blue), and symbol shapes indicate lake type (1–6; Nedbalová et al. 2013). Only those significant variables retained through backwards selection are plotted.

Finally, we sought to find the most parsimonious combination of variables explaining the observed patterns in bacterial and diatom communities among our lakes through the dbRDA models based on Bray–Curtis distances. For bacterial communities, pH (*F* = 2.92, *P* = .001), water temperature (2.41, 0.001), and conductivity (1.52, 0.015) showed the strongest association with the first two axes explaining a total of 17.7% of the variation (*F* = 2.28, *P* = .001; Fig. 7A). For diatom communities, significant variables included conductivity (*F* = 2.66, *P* = .001) and lake area (2.01, 0.026), with axes explaining 19.8% of the variation (Fig. 7B).

## Discussion

In Antarctica, regional climate warming and associated glacier recession is likely to lead to physico-chemical changes to freshwater ponds and lakes, as well as the creation of entirely new water bodies. Yet, it is unclear how these changes may influence the regional diversity and community structure of Antarctic freshwater microbial communities. In this work, we investigated how lake age and a gradient of associated environmental characteristics influence microbial community diversity and structure on Ulu Peninsula, JRI, Antarctic Peninsula. We found that old lakes hosted enhanced diversity and distinct communities compared to young lakes, which is likely due to contrasting chemical and physical characteristics (usually associated with lake ontogeny) between the different lakes rather than exclusively lake age. Nonetheless, our results suggest that in terms of climate change outcomes, newly created lakes do not guarantee comparable microbial habitat as old ones, and future work should continue disentangling drivers of regional community structure and diversity.

### Environmental characteristics and community structure between lake age groups

The results of the PCA suggest that, in general, young lakes have lower water temperatures, conductivities, and DOC concentrations, and are found at higher elevations with greater concentrations of P. Lower P and higher DOC concentrations in old lakes likely indicate greater primary producer activity (through uptake of the former and cell lysis/exudates of primary producers for the latter) than in young lakes, in line with the often prominent cyanobacterial mats in these former lake types. Overall, these patterns align well with Nedbalová et al. (2013), suggesting that these differences are probably stable over interannual timescales. In the case of temperature and elevation, these group differences are to be expected, given that the same conditions contributing to later glacier recession (geomorphology, shading, and so on) and consequently more recent lake formation, presumably also promote lower water temperatures in young lakes, along with the often persisting influx of glacier meltwater. In the future, new Ulu Peninsula waterbodies are expected to be located inland of existing ones (i.e. where most ice volume currently is), meaning that new lakes are likely to resemble the young lakes investigated here—cold, unstable, at higher elevations, and with lower conductivities than the already established, more coastal lakes.

From our sequencing of the 16S rRNA gene, we found that cyanobacterial reads were among the most detected, and dominated by filamentous *Phormidium, Leptolyngbya*, and *Tychonema*. This is consistent with work from other studies of Antarctic freshwaters (e.g. Ramoneda et al. 2021) and is intuitive given the nature of cyanobacteria-based microbial mats (Vincent et al. 1993). Yet, through plastidial 16S sequences, we also inferred the presence of eukaryotic primary producers dominated by diatoms, which have received focused investigations in this region in the past (Kopalová et al. 2013, 2019), as well as a *Nannochloropsis*-like group. These results are unlikely influenced by the issue of different per-cell numbers of chloroplasts across taxonomic groups as the detected diatoms typically possess two while *Nannochloropsis* one (e.g. Simionato et al. 2013). Nevertheless, the other issues (see the section “Methods”) may remain and caution is appropriate. The *Nannochloropsis*-like species are particularly interesting given their high inferred relative abundance and patchy distribution. The four lakes where it reached >75% of the relative abundance in the communities of microeukaryotic primary producers (i.e. VO2, MON, RO1, and FED) are spread throughout the Ulu Peninsula, and there is no obvious connection with either known environmental parameters or abundances of cyanobacteria. *Nannochloropsis* has been observed in Antarctic lakes before (Bielewicz et al. 2011, Karlov et al. 2017), and in other aquatic systems it can form blooms during cold-water periods (Krienitz et al. 2000, Fietz et al. 2005, Fawley and Fawley 2007). Given that DNA does not necessarily reflect physiologically active members of the community, it is possible that our observations reflect past *Nannochloropsis* blooms whose DNA was deposited in the sediments.

In general, the separation of old/young water bodies was remarkably clear in our analyses of microbial diversity and community structure (for both bacteria and diatoms), with greater diversity observed in the old lakes. While more work is certainly necessary to fully understand the mechanisms responsible for these differences, the first, and arguably the most obvious in the context of our study, is the difference in lake age itself: older water bodies may accumulate more diversity simply due to more time in existence. However, lake age is not an isolated variable, and encompasses different levels of both hydrological and geomorphological stability, solute concentrations, and thermal regimes. With this in mind, and given the results from model selection and field observations, we propose several potential explanations for the differences in community structure, including biological, physical, and chemical factors.

### Dispersal, environmental filtering, and biotic interactions

Given that lake age is the main variable tested for benthic microbial community diversity and structure in this study, it is tempting to speculate that the greater richness in old lakes could, at least in part, be attributed to the “slow and steady” accumulation of species over time, particularly in old lakes. Also in Antarctic soils, richer microbial communities were found in the areas deglaciated for a longer time, the suspected mechanisms here, however, include differentiation of soil strata due to a prolonged microbial activity (Almela et al. 2021). Likewise, in benthic microbial mats, given the continuous growth of mats on the surface sediments, and the eventual burial and/or decomposition of older growth, it may be erroneous to conceptualize these dynamic communities as passively gathering organisms over time. Furthermore, given the already “advanced” age of the young lakes (i.e. decades to a few centuries), coupled with the propensity of aeolian material to disperse in this region (Ambrožová et al. 2020, Kavan et al. 2020) and the relatively small spatial scale under consideration (ca 250 km^2^; Jennings et al. 2021), we predict that dispersal should not be limiting to the microbial communities on the Ulu Peninsula. While dispersal can of course be influential for microbial communities in Antarctica at large spatial scales (Sokol et al. 2013, Sakaeva et al. 2016, Schulte et al. 2022), preliminary analyses linking microbial diversity and community dissimilarity to spatial variables have thus far found no patterns indicating dispersal limitation on the Ulu Peninsula (Kavan, unpublished).

It is likely that lentic microbial mats are especially prone to filtering (either by environmental factors or biotic interactions) given their fixed position over the sediments and relatively high biomass (imparting greater community stability than in more dynamic habitats), making it difficult for potential colonists to establish. Indeed, visually different macroscopic mat forms are observed even in neighboring lakes (L. Nedbalová, personal observation) at scales where dispersal is not likely to be limiting. This is epitomized by the “old” lake Vondra 4 (VO4) located within a cluster of hydrologically connected “old” waterbodies to the south of the Ulu Peninsula (Fig. 1D). Despite this connectivity, it has strikingly different diversity values and benthic community structure compared to its neighbors, reflecting differences in physicochemical variables (most notably temperature). Thus, we hypothesize that environmental filtering and/or biotic interactions likely outweigh dispersal as a community structuring mechanism in the Ulu Peninsula lakes.

### Water temperature

Lake VO4 was a repeated exception to the overall clarity between lake age categories, consistently appearing among the young lakes in the ordinations despite being classified as “old” (Figs 2, 6A, and 7A). Revisiting field notes and photographs, it was revealed that VO4 was “90% frozen” at the time of sampling, and many young lakes have at least some ice cover in the photographs as well. In agreement, the VO4 water temperature measured at the time of sampling (which exceeds the average sampling time only by ca 10 min; see Note S2, Supporting Information, and Table 1 for more details) was 2°C, while an average of the measured water temperatures for the rest of the old lakes was 7.7 ± 3.5°C. In comparison, young lakes averaged at 1.7 ± 1.5°C (or 1.2 ± 0.6°C when the BLU young lake outlier with 5.5°C is excluded). In addition, in terms of pH (9.1) and specific conductivity (45 $\mu$S cm^−1^), VO4 did not differ considerably from the averages based on the rest of the old lakes (8.5 ± 0.7 and 212 ± 224 $\mu$S cm^−1^, respectively). Thus, water temperature and/or ice cover may be an influential variable for community structuring (perhaps even overriding other influences). This is further supported by our linear modeling, which identified water temperature as a significant factor for both bacterial and diatom richness.

Indeed, colder temperatures may directly select for cryophilic taxa, placing another environmental filter on the regional flora to establish in the area. For example, young lakes were characterized by ASVs from genera *Polaromonas, Pedobacter, Luteolibacter*, and *Sphingomonas* (among others). Interestingly, these four genera were all highlighted in a recent metastudy by Bourquin et al. (2022) as being disproportionately represented in the cryosphere. The anomalous lake VO4 in particular had higher relative abundances of ASVs identified to the genus *Pedobacter* (i.e. 2.7% compared to the other old lakes averaging at 0.5%), and *Pedobacter* was also identified by ANCOM as being differentially more abundant in the young lakes, where it was observed at an average abundance of 2.0%. Thus, even within an already cryospheric ecosystem (i.e. coastal Antarctica), these young lakes were arguably “more cryospheric” in terms of their community structure than their older counterparts.

It must be noted that inter- and intraseasonal water temperature fluctuations are an important caveat when interpreting our results, as water temperatures on JRI can vary considerably over time and between lake types (Nedbalová et al. 2013, Kavan 2017, Roman et al. 2019, Kavan et al. 2021). These fluctuations are typically due to diel and meteorologically induced changes in solar radiation, and would require a full season of littoral temperature measurements from each lake to adequately characterize. Nevertheless, potentially different meteorological conditions on different sampling days and times are highly unlikely to cause the observed differences in water temperatures between old and young lake groups (i.e. 7.7 ± 3.5°C and 1.2 ± 0.6°C, respectively) since all lakes were sampled during a narrow range of dates (8–17 February 2017) with both young and old lakes typically sampled on the same day (Table 1), and in comparable sampling times (Table S2 and Note S2, Supporting Information).

### Stability and conductivity

As discussed by Nedbalová et al. (2013), lake age on the Ulu Peninsula is linked with habitat stability. While “habitat stability” is a difficult variable to quantify given multiple contributing factors and mostly anecdotal observations due to a lack of long-term data (and thus difficult to test for), both hydrological and geomorphological factors are likely candidates for the differences in observed benthic diversity and community structure. Furthermore, different lake types are subject to different kinds of instability. For example, while old lakes demonstrate more permanence due to the greater geomorphological stability of their surroundings, they are also more prone to periodic drying (either intra- or interannually) due to being shallower. This drying may lead to the periodic desiccation of the microbial mats. Interestingly, since diatoms are routinely used as bioindicators of hydrological conditions in Antarctica and beyond (e.g. Spaulding et al. 2010), they may provide evidence of such periodic desiccation. Indeed, putatively desiccation tolerant diatom taxa such as *P. australomicrostauron* and *H. amphioxys* were listed as indicators of old lakes, while other desiccation-resistant diatoms (e.g. *Achnanthes coarctata, Pinnularia borealis*; Souffreau et al. 2010, 2013, Tofilovska et al. 2014) were completely absent in young lakes. These results broadly conform to our expectations, given that more desiccation tolerant taxa should be found in habitats where desiccation is more likely (i.e. old lakes). Yet, an alternative explanation is offered. Given that these diatom species are primarily aerophilic and/or semiterrestrial (e.g. Kopalová et al. 2014, Chattová et al. 2022), more developed terrestrial soils and vegetation in the catchment may be necessary for their transport into, and ultimate establishment within, corresponding waterbodies. Such sources may be simply lacking in more recently deglaciated higher-lying surroundings of younger lakes, yet the influence of these adjacent communities on the lake mat diatoms remains to be tested.

In contrast, young lakes feature geomorphologically unstable margins with steeper slopes, which may discourage the accumulation of mat biomass and growth. Some of these young lakes may eventually drain with the collapse of supporting structures (e.g. ice dams) leading to lake outburst events, as was recently documented here for a kettle lake (Sone et al. 2007). As such, many of the young lakes may eventually disappear with further melting, despite their recent formation. At the same time, lower temperatures found in young lakes also promote increased ice cover, which may reduce levels of photosynthetically active radiation, creating light-limiting conditions for photoautotrophs, and potentially creating energy-limiting conditions for heterotrophs (Rochera et al. 2010). Furthermore, the prolonged inundation of ice at young lake margins may reduce the amount of suitable mat habitat, further preventing the establishment of the same community types found in old lakes. While cyanobacterial relative abundances were not significantly different between young and old lakes (although this would likely differ if absolute abundances were measured), the results of the indicator species analysis showed that ASVs most strongly associated with the old lakes were mat-forming cyanobacteria such as *Leptolyngbya* and *Pseudoanabaena*. This may be unsurprising, given that the old lakes often host more visually extensive mats than young lakes. On the other hand, some cyanobacterial ASVs (i.e. *Chamaesiphon*) were also found to be significant indicators of the young lakes. Therefore, cyanobacteria in the area exhibit complex eco-evolutionary patterns, with some species/strains potentially better adapted to the less stable conditions of the young lakes, while others thrive in those of the old.

A final consideration is electroconductivity (and perhaps by extension pH, though this can vary intra- and interannually as for the temperature, and should be interpreted cautiously), which was situated along axis 1 of the PCA (being higher in old lakes and lower in young lakes), and among the most influential variables to both diatom and bacterial communities. Given the periodic desiccation of the old lakes, their proximity to the coast, as well as a greater number of accumulated seasons to accumulate solutes, their waters tend to be more concentrated with ions. Thus, it is possible that greater ionic concentrations (which may be derived from marine aerosols) can provide more opportunities for energy acquisition and biomass accumulation (and therefore more DOC, creating a positive feedback loop), as well as promote more and diverse ecological niches, and in turn increase diversity. Conductivity was previously found to be an important predictor of diatom community structure on the Ulu Peninsula (Kopalová et al. 2013) as well as in epilithon on the nearby Clearwater Mesa (Kopalová et al. 2019). Likewise, it was found to significantly influence the structure of lacustrine benthic bacterial communities throughout Antarctica (Peeters et al. 2011). Yet, microbial mats are also notorious for creating their own microhabitat conditions within the mat matrix (Villeneuve et al. 2001, Mueller and Vincent 2006), concentrating important elements for communal use. Therefore, future work is necessary to fully disentangle the importance of solute availability, along with the thermal and physical constraints outlined above.

## Conclusions

Little is known about the microbial community structure and ecology of smaller lakes and ponds in Antarctica, and especially on the Antarctic Peninsula. Here, we investigated the microbial communities of 29 lakes on the Ulu Peninsula, JRI, one of the largest glacier-free areas of Antarctica. We observed that communities differed between the two age classes, and the young lakes hosted substantially less diversity on average than older lakes. This means that despite an increasing freshwater habitat area on the Antarctic Peninsula, the new waterbodies are not likely to host the same microbial communities as the established ones. While the exact mechanism for these differences was not conclusively elucidated, we argue that lake age itself is unlikely to be directly responsible. Instead, contrasting physical and chemical factors associated with individual lakes histories, including temperature regime, solute concentrations, and hydrological and geomorphological stability, are likely the most influential. Overall, this work represents a useful contribution to further our understanding of climate-related changes to diversity on the Antarctic Peninsula, and will point us in the right direction in terms of how best to prioritize future conservation efforts in the area.

## Supplementary Material

fiad087_Supplemental_File
