# Supplementary material for: Recently formed Antarctic lakes host less diverse benthic bacterial and diatom communities than their older counterparts
Source: FEMS Microbiol Ecol. 2023 Jul 29;99(9):fiad087. doi: 10.1093/femsec/fiad087 (PMC10446143; doi:10.1093/femsec/fiad087)
Supplement: fiad087_Supplemental_File [file fiad087_supplemental_file.pdf]

Supplementary information to the article

Recently-formed Antarctic lakes host less diverse benthic bacterial and diatom communities than their older counterparts

by

Jan Kollár, Kateřina Kopalová, Jan Kavan, Kristýna Vrbická, Daniel Nývlt, Linda Nedbalová, Marek Stibal and Tyler J. Kohler

Contents

Note S1. On the ages of the JRI lakes

Note S2. On the water temperature and sampling times

Figure S1. Alpha rarefaction curves for the 29 bacterial communities based on the Observed ASVs

Table S1. Hydrochemistry of the lakes

Table S2. Comparison of old and young lake groups in selected parameters

Table S3. Different diversity metrics for bacterial and diatom communities of 'old' and 'young' lakes

Note S1. On the ages of the JRI lakes

In general, the geomorphological evolution (e.g., Davies et al., 2013; Jennings et al., 2021) and the obtained deglaciation chronologies (e.g., Johnson et al., 2011; Glasser et al., 2014; Nývlt et al., 2014 including unpublished data of Daniel Nývlt, which are currently being put together for two publications), as well as the ages of the origin of some of the lakes (e.g., Bjorck et al., 1996; Hjort et al., 1997; Píšková et al., 2019; Čejka et al., 2020 and further unpublished data from Monolith Lake of Daniel Nývlt, which are currently being put together for a publication) show a rather simple geomorphological and deglaciation history of the currently deglaciated parts of James Ross Island (JRI).

The deglaciation of the lowest lying parts of northern James Ross Island started during the Termination I (Pleistocene-Holocene transition), and coastal zones became glacier-free by 12.9 ka ago (Nývlt et al., 2014). This is the area of Lachman lakes (LA1 and LA2), and the dating of basal lacustrine sediments suggests their origin to be approximately 11.9 ka ago (Hjort et al., 1997). These are by far the oldest lakes on the island, even though they are rather shallow and may dry out during some summer seasons. The rapid early deglaciation during the Holocene lead to the splitting of local glacier cover on James Ross Island from the Antarctic Peninsula Ice Sheet and local glaciers behaved independently since the early Holocene (Glasser et al., 2014; Nývlt et al., 2014, 2020). The climatic conditions of most of the Holocene were very similar for the current climate (average for 1950–2000 CE) as calculated by the temperature anomalies from the James Ross Island Ice Cap (Mulvaney et al., 2012). This led to a slow glacier recession of local glaciers between 8 and 2 ka ago (Glasser et al., 2014; unpublished data of Daniel Nývlt) to an extent smaller than at present (Nývlt et al., 2020). The only prominent cooling leading to the Neoglacial phase of local glaciers advances began in this area 2.5–2.0 ka ago (Sterken et al., 2012; Mulvaney et al., 2012; Čejka et al., 2020). Local glaciers advanced from their accumulation areas and deposited prominent frontal and lateral moraines during the Neoglacial phase. The advance culminated approx. 1.0–0.8 ka ago with a second less prominent advance 0.4–0.3 ka ago as evidenced from the Lookalike Glacier (unpublished data of Daniel Nývlt). Since 0.3–0.2 ka ago, all local glaciers retreated with a prominent speedup during the last decades as evidenced by the studies of Carrivick et al. (2012), Engel et al. (2012), and Kaplan et al. (2020).

Basing on the geomorphological evolution outlined above, the lakes which are located outside of the Neoglacial moraines (i.e. our “old” lakes) must be older than 2.0 ka, and some of them are likely even older as evidenced by the dating of basal sediments in the Lachman Lakes. On the contrary, lakes associated with the deglaciation after the Neoglacial culmination (i.e. our “young” lakes) must be younger than approx. 300 years, some of them evolving only during the last decades. The youngest lakes on the Peninsula are kettle lakes, the origin of which could be seen directly in the field evolving in the Neoglacial moraines of local glaciers in connection with the deepening of lakes due to the thermal effect of freshwater on the underlying ice, which still forms the largest proportion of the moraines. A glacier lake outburst of one such kettle lake was recently documented by a Japanese team (Sone et al., 2007).

References:

Björck S, Olsson S, Ellis-Evans C *et al.* Late Holocene palaeoclimatic records from lake sediments on James Ross Island, Antarctica. *Palaeogeogr Palaeoclimatol Palaeoecol* 1996;**121**(3-4):195-220.

Carrivick JL, Davies BJ, Glasser NF *et al.* Late-Holocene changes in character and behaviour of land-terminating glaciers on James Ross Island, Antarctica. *J Glaciol* 2012;**58**(212):1176-1190.

Čejka T, Nývlt D, Kopalová K *et al.* Timing of the neoglacial onset on the North-Eastern Antarctic Peninsula based on lacustrine archive from Lake Anónima, Vega Island. *Glob Planet Change* 2020;**184**: DOI: 10.1016/j.gloplacha.2019.103050.

Davies BJ, Glasser NF, Carrivick JL *et al.* Landscape evolution and ice-sheet behaviour in a semi-arid polar environment: James Ross Island, NE Antarctic Peninsula. *Geol Soc Spec* 2013;**381**:353-395.

Engel Z, Nývlt D, Láska K *et al.* Ice thickness, areal and volumetric changes of Davies Dome and Whisky Glacier (James Ross Island, Antarctic Peninsula) in 1979–2006. *J Glaciol* 2012;**58**:904-914.

Glasser NF, Davies BJ, Carrivick JL *et al.* Ice-stream initiation, duration and thinning on James Ross Island, northern Antarctic Peninsula. *Quat Sci Rev* 2014;**86**:78-88.

Hjort C, Ingólfsson Ó, Möller P *et al.* Holocene glacial history and sea-level changes on James Ross Island, Antarctic Peninsula. *J Quat Sci* 1997;**12**:259–273.

Jennings SJA, Davies BJ, Nývlt D *et al.* Geomorphology of Ulu Peninsula, James Ross Island, Antarctica. *J Maps* 2021;**17**:125-139.

Johnson JS, Bentley MJ, Roberts SJ *et al.* Holocene deglacial history of the northeast Antarctic Peninsula – a review and new chronological constraints. *Quat Sci Rev* 2011;**30**(27):3791-3802.

Kaplan MR, Strelin JA, Schaefer JM *et al.* Holocene glacier behavior around the northern Antarctic Peninsula and possible causes. *Earth Planet Sci Lett* 2020;**534**(A):116077.

Mulvaney R, Abram NJ, Hindmarsh RCA *et al.* Recent Antarctic Peninsula warming relative to Holocene climate and ice-shelf history. *Nature* 2012;**489**: 141–144.

Nývlt D, Braucher R, Engel Z *et al.* Timing of the Northern Prince Gustav Ice Stream retreat and the deglaciation of northern James Ross Island, Antarctic Peninsula during the last glacial–interglacial transition. *Quat Res* 2014;**82**:441–449.

Nývlt D, Glasser NF, Hocking EP *et al.* Tracing the deglaciation since the Last Glacial Maximum. In: Oliva M, Ruiz-Fernández J (ed.). Past Antarctica. *Academic Press* 2020: 89-107.

Píšková A, Roman M, Bulínová M *et al.* Late-Holocene palaeoenvironmental changes at Lake Esmeralda (Vega Island, Antarctic Peninsula) based on a multi-proxy analysis of laminated lake sediment. *Holocene* 2019;**29**:1155–75.

Sone T, Fukui K, Strelin JA *et al*. Glacier lake outburst flood on James Ross Island, Antarctic Peninsula region. *Pol Polar Res* 2007;**28**:3–12.

Sterken M, Roberts SJ, Hodgson DA *et al.* Holocene glacial and climate history of Prince Gustav Channel, northeastern Antarctic Peninsula. *Quat Sci Rev* 2012;**31**: 93-111.

Note S2. On the water temperature and sampling times

In our analyses, water temperature consistently appeared among the variables potentially contributing to the observed variation between microbial communities of the old and young lakes. An average of the measured water temperatures for the old lakes (excluding the anomalous VO4 - see Discussion for more details) was 7.7 ± 3.5 °C while the young lakes averaged at 1.7 ± 1.5 °C (or 1.2 ± 0.6 °C when BLU young lake outlier with 5.5°C is excluded). Although water temperature (as well as pH in response to a rate of photosynthesis) typically changes with daytime (and weather conditions – see below), our sampling times were not drastically different between the lake groups (with medians ± standard deviations of 15:45 ± 70 min and 16:30 ± 165 min of the local time for old and young lakes, respectively).

While we lack information on the weather/air temperature/sunlight conditions on the sampling days (between 8th and 17th February 2017), lakes from both old and young groups were typically sampled on the same day (e.g., 5 old lakes and 2 young on the 12th of February, 5 old and 2 young on the 13th of February, 2 old and 4 young on the 15th of February; Table 1). Moreover, the rogue lake VO4 was sampled on the 9th of February along with 5 other old lakes, all six between 14:20 and 16:40). Therefore, in the case of this study, potentially different weather conditions on different sampling days and times seem highly unlikely to cause the observed differences between old and young lake groups.

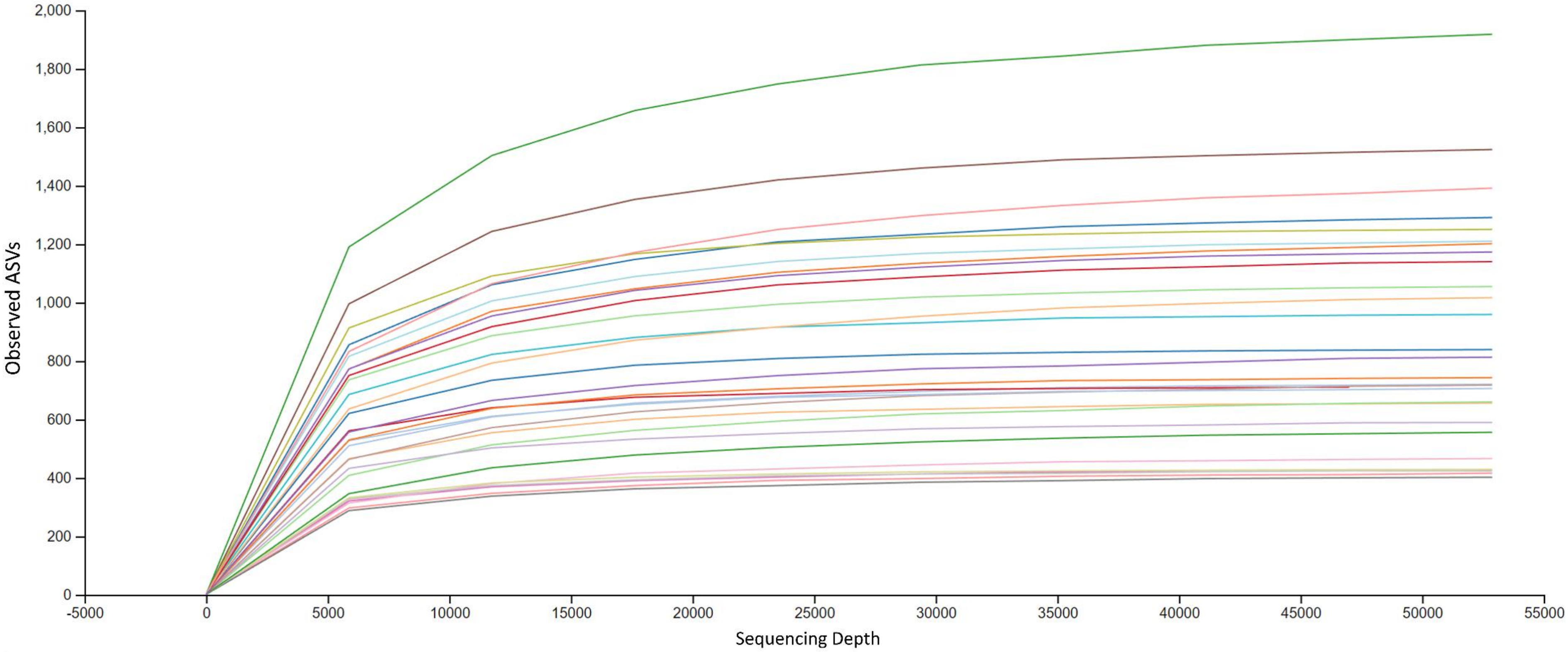

Figure S1. Alpha rarefaction curves for the 29 bacterial communities based on the Observed ASVs. Shannon Diversity revealed the same (not shown).

Table S1. Hydrochemistry of the lakes. Samples analyzed for diatoms are indicated by an asterisk (n.a. = not available).

Lake	SO_4^{2-} [mg l^{-1}]	N-NH_4^+ [mg l^{-1}]	N-NO_3^- [mg l^{-1}]	N_{tot} [mg l^{-1}]	Cl^- [mg l^{-1}]	F^- [mg l^{-1}]	DOC [mg l^{-1}]	P_{tot} [mg l^{-1}]	Na^+ [mg l^{-1}]	K^+ [mg l^{-1}]	Ca^{2+} [mg l^{-1}]	Mg^{2+} [mg l^{-1}]
VO1*	1.38	< 0.039	< 0.113	< 1.00	3.45	< 0.100	2.10	< 0.025	6.10	< 1.00	2.560	1.030
VO4*	1.99	< 0.039	< 0.113	< 1.00	3.30	< 0.100	1.10	0.058	4.08	< 1.00	3.100	0.874
WH1*	14.20	< 0.039	< 0.113	< 1.00	18.60	< 0.100	2.20	< 0.025	12.70	< 1.00	5.250	3.540
GR1*	3.95	0.061	< 0.113	< 1.00	22.40	< 0.100	2.50	< 0.025	13.30	< 1.00	5.040	2.790
GR2*	5.96	0.060	< 0.113	< 1.00	33.70	< 0.100	3.10	< 0.025	19.00	1.40	10.300	5.280
RED	7.18	0.088	< 0.113	< 1.00	72.40	< 0.100	2.80	< 0.025	31.30	1.36	10.400	7.980
CYA*	1.30	< 0.039	< 0.113	< 1.00	3.35	< 0.100	1.00	0.077	6.90	< 1.00	5.900	0.801
BLA	2.00	< 0.039	< 0.113	< 1.00	13.70	< 0.100	3.80	< 0.025	8.87	1.43	13.100	4.010
VO3*	0.78	< 0.039	< 0.113	< 1.00	2.95	< 0.100	1.40	< 0.025	5.46	< 1.00	2.250	0.916
VO2*	1.38	< 0.039	< 0.113	< 1.00	4.54	< 0.100	1.20	0.028	5.67	< 1.00	3.780	1.000
LA2*	83.20	0.318	< 0.113	< 1.00	91.90	< 0.100	3.70	< 0.025	46.70	3.51	22.400	20.800
LA1*	205.00	0.150	< 0.113	< 1.00	199.00	< 0.100	6.20	0.031	107.00	6.32	44.200	38.300
MUD	135.00	0.047	< 0.113	< 1.00	37.80	< 0.100	2.90	< 0.025	40.00	4.02	32.000	7.470
PHO*	95.90	< 0.039	< 0.113	< 1.00	15.20	< 0.100	1.50	< 0.025	21.60	< 1.00	26.700	5.440
MON-C*	12.00	0.070	< 0.113	< 1.00	10.30	< 0.100	2.00	< 0.025	8.15	< 1.00	9.500	1.760
KA2*	36.70	0.103	< 0.113	< 1.00	23.50	< 0.100	3.20	< 0.025	15.70	< 1.00	24.600	3.660
DAN	37.70	< 0.039	< 0.113	< 1.00	8.41	< 0.100	1.50	< 0.025	12.40	< 1.00	17.700	2.540
LOU*	229.00	0.143	< 0.113	< 1.00	24.20	0.123	2.20	< 0.025	44.00	1.21	82.900	11.500
KA1	26.30	0.109	< 0.113	< 1.00	21.00	< 0.100	2.70	< 0.025	12.10	< 1.00	16.600	2.970
BIB*	1.51	< 0.039	< 0.113	< 1.00	6.84	< 0.100	0.98	0.092	4.92	2.53	0.860	0.393
FED	2.21	0.042	< 0.113	< 1.00	7.48	< 0.100	1.40	0.073	9.72	< 1.00	3.740	0.961
BIB-2	1.51	< 0.039	< 0.113	< 1.00	6.84	< 0.100	0.98	0.092	4.92	2.53	0.860	0.393
RO1*	5.88	0.041	< 0.113	< 1.00	7.13	< 0.100	1.30	0.127	15.50	< 1.00	1.690	0.445
NAD*	5.99	0.056	< 0.113	< 1.00	14.40	< 0.100	1.50	< 0.025	9.02	1.01	2.350	1.760
BLU*	4.58	0.110	< 0.113	< 1.00	8.80	< 0.100	1.80	0.054	10.20	< 1.00	8.560	0.993
RO2*	5.34	0.049	< 0.113	< 1.00	14.00	< 0.100	1.50	0.070	13.20	< 1.00	1.880	0.935
OM1*	1.88	< 0.039	< 0.113	< 1.00	6.90	< 0.100	1.50	0.128	11.40	< 1.00	0.666	0.390
GIN*	n.a.	n.a.	n.a.	n.a.	n.a.	n.a.	n.a.	n.a.	n.a.	n.a.	n.a.	n.a.
LAS*	3.65	< 0.039	< 0.113	< 1.00	10.40	< 0.100	1.10	0.035	9.74	< 1.00	2.380	0.939

Table S2. Comparison of old and young lake groups in selected parameters. Values of minimum, maximum, median ± standard deviation, first and third quartiles are shown. Results incorporating the 1.5×IQR outliers are shown where detected. Since water temperature and pH may be indirectly influenced by daytime, local sampling time is included to demonstrate that lakes from the old and young groups were not sampled during considerably different daytimes. See Note S2 for arguments on why sampling times and dates are unlikely to influence the differences between old and young lake groups observed in this study. The information about lake volume, mean depth and maximal depth was extracted from Nedbalová et al. (2013).

	old					young				
	MIN	Q1	MED ± STD	Q3	MAX	MIN	Q1	MED ± STD	Q3	MAX
**Excluding outliers:**										
Altitude [m a.s.l.]	4	37	65 ± 86	176	184	184	201	250 ± 31	257	269
Lake volume [m^3]	195	1,277	2,183 ± 5,855	6,593	18,220	1,824	5,120	7,880 ± 17,784	7,880	45,050
Mean depth [m]	0.1	0.2	0.5 ± 0.5	1.3	1.5	1.1	1.1	1.3 ± 1.9	2.6	5.5
Maximal depth [m]	0.2	0.4	1.1 ± 1.3	2.2	3.9	2.7	2.7	3.8 ± 3.9	4.6	12.0
pH	7.2	7.9	8.8 ± 0.7	9.2	9.6	8.1	8.8	9.2 ± 0.5	9.2	9.6
Conductivity [$\mu S\ cm^{-1}$]	45	83	128 ± 112	198	411	29	37	58 ± 22	72	91
Temperature [°C]	2	5.3	7.1 ± 3.5	9.6	15.7	0.6	0.9	1.1 ± 0.6	1.4	2.1
Sampling time	9:40	13:55	15:45 ± 70 min	18:00	19:15	16:00	16:00	16:30 ± 165 min	17:30	19:30
**Including outliers (where applicable):**										
Altitude [m a.s.l.]						184	210	250 ± 57	259	387
Lake volume [m^3]	195	1,418	4,510 ± 27,775	8,750	116,600	1,824	5,810	7,880 ± 639,698	35,758	1,580,000
Mean depth [m]						1.1	1.2	2.0 ± 6.6	4.8	17.9
Maximal depth [m]						2.7	3.0	4.2 ± 11.7	10.2	32.5
pH						7.8	8.7	9.2 ± 0.7	9.3	10.4
Conductivity [$\mu S\ cm^{-1}$]	45	84	142 ± 224	267	948					
Temperature [°C]						0.6	0.9	1.3 ± 1.5	1.9	5.5

Table S3. Different Alpha diversity metrics' means ± standard deviations for bacterial and diatom communities of 'old' and 'young' lakes. Kruskal-Wallis test's H and p values are given and significance at α < 0.05 is indicated by an asterisk (n.a. = not available). References for the metrics may be found in the main body of the article.

	bacteria				diatoms			
	old	young	H	p	old	young	H	p
Observed ASVs/Species	1052.9 ± 340.4	523.7 ± 146.5	15.57	< 0.001*	19.4 ± 5.5	10.0 ± 4.5	10.57	0.001*
Shannon's Diversity Index	7.555 ± 0.677	6.015 ± 0.966	12.81	< 0.001*	2.833 ± 0.727	1.592 ± 0.560	10.73	0.001*
Pielou's Evenness	0.757 ± 0.042	0.669 ± 0.098	6.14	0.013*	0.663 ± 0.128	0.502 ± 0.123	6.38	0.012*
